# Synthesis and spectroscopic properties of (N/O) mono- and dispirocyclotriphosphazene derivatives with benzyl pendant arms: study of biological activity *This manuscript is dedicated to Prof. Dr. Adem Kılıç on his retirement.


**DOI:** 10.3906/kim-1908-17

**Published:** 2020-02-11

**Authors:** Özlem İŞCAN, Reşit CEMALOĞLU, Nuran ASMAFİLİZ, Zeynel KILIÇ, Leyla AÇIK, Pelin ÖZBEDEN, Tuncer HÖKELEK

**Affiliations:** 1 Department of Chemistry, Faculty of Science, Ankara University, Ankara Turkey; 2 Department of Biology, Faculty of Science, Gazi University, Ankara Turkey; 3 Department of Physics, Faculty of Science, Hacettepe University, Beytepe, Ankara Turkey

**Keywords:** Pendant-armed spirocyclotriphosphazenes, crystal structure, spectroscopy, antimicrobial activity, DNA interaction

## Abstract

The Cl replacement reactions of hexachlorocyclotriphosphazene (trimer; N _3_ P _3_ Cl _6_ ) with sodium (N-benzyl)- aminopropanoxides (1 and 2) produced monospiro- (3 and 4), cis-, and trans-dispirocyclotriphosphazenes (13–16). The monospiro tetrakis-aminocyclotriphosphazenes (5–12) were obtained by the Cl substitutions of 3 and 4 with different secondary amines. The cis- (13 and 14) and trans-dispirophosphazenes (15 and 16) possessed 2 chiral P centers, and they were able to present meso and racemic forms, respectively. Moreover, the structures of compounds 5 and 14 were designated using X-ray data. The absolute configuration of compound 14 was found as SR in the solid state. Analytical and spectroscopic data of the phosphazenes were consistent with their suggested structures. Antimicrobial activities of the benzyl-pendant-armed cyclotriphosphazenes were scrutinized against G(+) and G(−) bacteria and yeast strains. The bacterium most affected by the synthesized compounds was *Pseudomonas aeruginosa* . Minimum inhibitory concentrations and minimal bacterial concentrations were in the range of 125–500 μM. Interactions between the phosphazenes (3–12 and 15) and plasmid DNA were studied with agarose gel electrophoresis. The phosphazene- DNA interaction studies of the cyclotriphosphazenes revealed that phosphazenes 3, 4, and 15 had a substantial effect on supercoiled DNA by cleavage of the double helix.

## 1. Introduction

Phosphazenes refer to phosphorus-nitrogen compounds that occur by the sequential bonding of atoms to each other [1,2]. Hexachlorocyclotriphosphazene (N _3_ P _3_ Cl _6_ , trimer) is versatile and has the ability to react easily with different mono-, di-, and multifunctional reagents [3–5]. When trimer is reacted with monofunctional agents, at least 6 different cyclotriphosphazene derivatives can be formed [6]. In addition, the Cl replacement reactions of trimer with one equimolar difunctional reagent may predominantly yield monospiro products when compared to other expected ansa- and bino-isomers [7,8]. Moreover, when two equimolar difunctional agents are used, dispirocyclotriphosphazenes with cis- and trans-geometrical and meso/racemic optical isomers can occur [9–11]. It is difficult to separate these isomers from each other; hence, very few optical and geometrical isomers of dispiro derivatives have been separated in the literature [9–13]. Geometric isomers of dispirocyclotriphosphazenes can be separated by column chromatography, while optical isomers may be determined using ^31^ P { ^1^ H}, circular dichroism, X-ray data, and/or high-pressure liquid chromatography [14–17]. Among these methods, X-ray data are important to define the absolute configurations of optically active phosphazenes.

On the other hand, phosphazene derivatives with different chemical properties can be utilized in various fields, such as technology, biochemistry, and medicine. Organocyclotriphosphazene derivatives are used as antimicrobial [18] and anticancer [19,20] agents, lubricants [21], flame retardant additives for organic polymers [22], chemosensors [23], and liquid crystals [24].

In the literature, some cis- and trans-dispirophosphazene products have been presented, but dispirocyclotriphosphazene derivatives with pendant arms are less common [25,26]. The isomer distributions, chiralities, and structural and spectral features of dispiro products with pendant arms were determined in these studies. In order to compare with the reports in the literature, (N/O) mono- and dispirocyclotriphosphazene derivatives with benzyl pendant arms were prepared in this study. Furthermore, the mono- and dispirocyclotriphosphazene derivatives reported here were also prepared for the determinations of their chemical and biological aspects.

## 2. Results and discussion

### 2.1. Synthesis

In synthesis, sodium (N-benzyl)aminopropanoxides 1 and 2 were resynthesized for the preparations of the monospirophosphazenes (3 and 4) obtained previously [27], as well as the syntheses of the new cis- and trans-dispirocyclotriphosphazenes (13–16). The Cl exchange reactions of 1 equimolar amount of sodium (Nbenzyl) aminopropanoxides (1 and 2) with 1 equimolar amount of N _3_ P _3_ Cl _6_ afforded the monospirophosphazenes with a benzyl pendant arm (3 and 4), according to the literature method. Compounds 3 and 4 possessed 4 exchangeable Cl atoms, owing to the Cl substitution reactions with secondary amines. Hence, tetrakispyrrolidino-(5 and 6) [27], morpholino- (7 and 8), piperidino- (9 and 10), and 1,4-dioxa-8-azaspiro[4,5]decane-(DASD)-phosphazenes (11 and 12) were obtained from the reactions of the corresponding monospirophosphazenes (3 and 4) with excess amounts of pyrrolidine, morpholine, piperidine, and DASD, respectively. Moreover, the condensations of 1 equimolar amount of N _3_ P _3_ Cl _6_ with 2 equimolar amounts of sodium(Nbenzyl) aminopropanoxides (1 and 2) yielded monospiro- (3 and 4) and cis- and trans-dispirocyclotriphosphazenes (13–16) (Scheme). The yields of the mono- (3 and 4), cis- (13 and 14), and trans-dispiro compounds (15 and 16) were 26%, 25%, 30%, 32%, 37%, and 37%, respectively. As understood, the yields of the trans-derivatives were larger than those of the mono- and cis-dispiro products. It could be suggested that the reason for the low yields of the cis-derivatives was the steric interactions between the benzyl groups. THF was used as a solvent in all of the substitution reactions. All of the phosphazene products were purified by column chromatography.

**Scheme:**
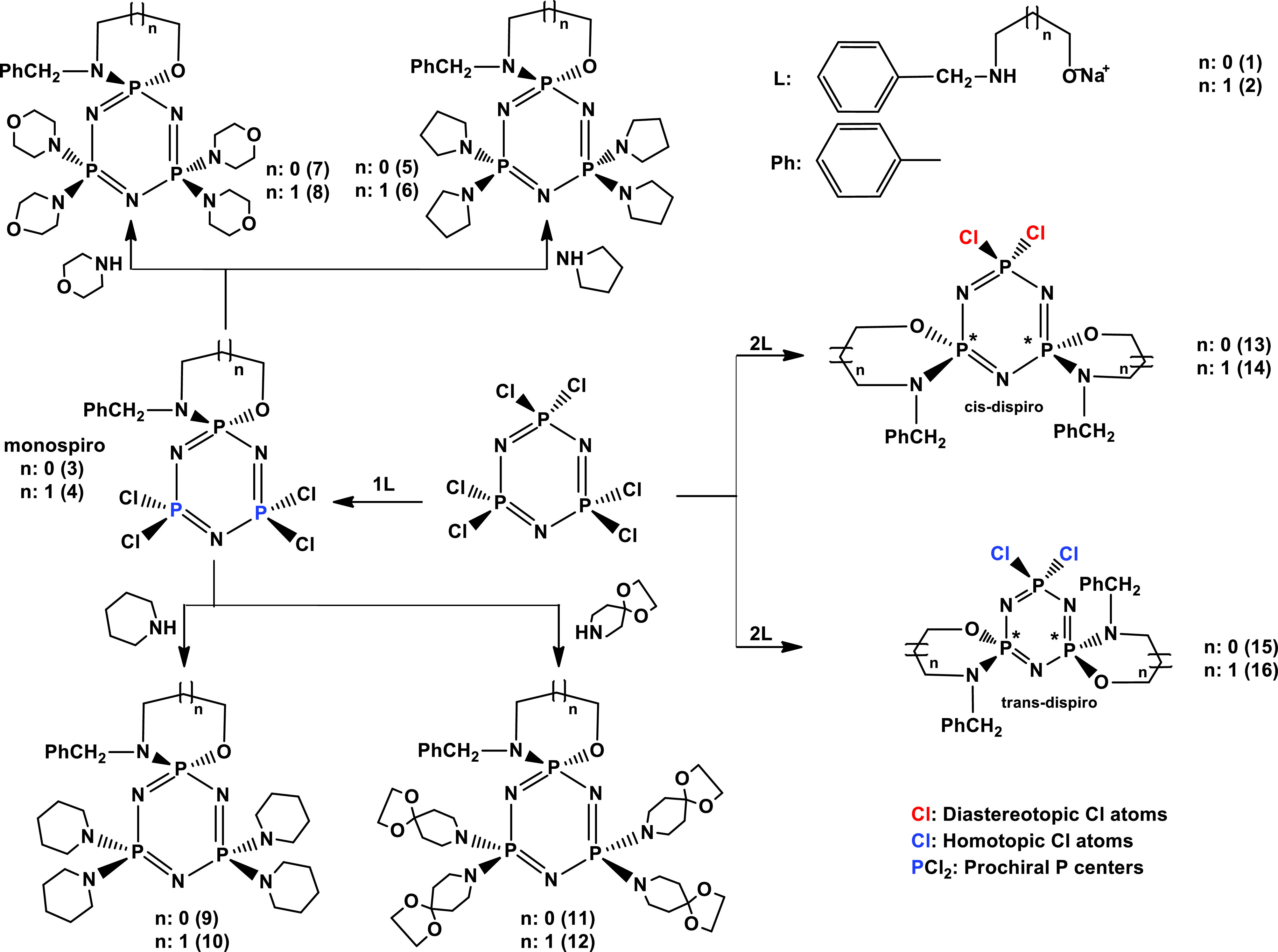
Replacement reactions of N _3_ P _3_ Cl _6_ with the monodentate and bidentate amines.

It has already been mentioned that phosphazenes 3–6 were presented in the literature [27]. According to the literature, phosphazenes 3 and 4 were synthesized in THF, while compounds 5 and 6 were synthesized in toluene. However, in the present study, the same compounds (3–6) were synthesized in THF. The reaction yields of 3–6 previously reported in the literature were 44%, 55%, 34%, and 45%, respectively [27]. As understood, the reaction yields in this study were higher than those given in the literature. For comparison, the yields of compounds 3–6 can be seen in Section 3.3. Recently, the syntheses of some cis- and transspirocyclotriphosphazenes with 4-chloro/4-nitro/4-fluoro-benzyl pendant arms were reported in the literature [28,29]. Although the trans-geometric isomers of these compounds were purely obtained, the cis-geometric isomers were not isolated from the reaction mixture using crystallization, column chromatography, or preparative TLC. Furthermore, the cis- and trans-isomers of dispirocyclotriphosphazenes with benzyl pendant arms were isolated by column chromatography in this study.

The cis- (13 and 14) and trans-phosphazenes (15 and 16) possessed 2 stereogenic P atoms, and they were probably present in the meso (RS/SR) and racemic (RR/SS) forms, respectively. As expected, cis-isomer 13 was present as a meso (SR) configuration.

The IR, APIES-MS, NMR, and microanalytical results were consistent with the suggested formulae of the mono- and dispirophosphazene derivatives. Protonated molecular ion peaks ([MH] ^+^ ) appeared for compounds 7–12, whereas molecular ion peaks ([MH] ^+^ emerged for compounds 13–16 in the mass spectra.

### 2.2. NMR and IR spectroscopies

The data obtained from the ^31^ P { ^1^ H} spectra of the mono- (3–12) and cis- and trans-dispirophosphazenes (13–16) are given in Table 1. The partly and fully substituted monospiro- (3–12) and dispirocyclotriphosphazenes (13–16) had AX _2_ spin systems. A triplet for 1 P atom and a doublet for 2 P atoms were determined in the spectra of the phosphazenes with AX _2_ spin systems. Moreover, the cis- and trans-dispiro products (13–16) had a triplet for 1 P atom and a doublet for 2 P (spiro) atoms. The chemical shifts and ^2^ J _*PP*_ values of benzylamino monospirophosphazenes containing 5-membered spiro precursors were larger than those of the other phosphazenes bearing 6-membered spiro-rings. Moreover, the average ^2^ J _*PP*_ constants were 48.4 and 46.2 Hz for the products, including the 5- and 6-membered spiro rings. These findings were inconsistent with the literature data [28,29].

**Table 1:** 31P NMR (decoupled) spectral data of the phosphazenes [chemical shifts (δ)are presented in ppm andJvalues in Hz]a.

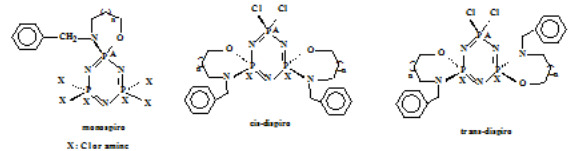					
Compound	Spin system	PCl2	P(NR)2	P(spiro)	2JP P
**3**	AX _2_	24.88 (d)	-	21.03 (t)	52.6
**4**	AX _2_	23.25 (d)	-	9.18 (t)	51.0
**5**	AX _2_	-	19.54 (d)	31.94 (t)	47.0
**6**	AX _2_	-	19.03 (d)	21.28 (t)	45.3
**7**	AX _2_	-	22.59 (d)	31.49 (t)	47.0
**8**	AX _2_	-	21.46 (d)	19.97 (t)	44.7
**9**	AX _2_	-	23.32 (d)	31.80 (t)	47.0
**10**	AX _2_	-	22.23 (d)	20.30 (t)	46.2
**11**	AX _2_	-	22.39 (d)	31.19 (t)	48.6
**12**	AX _2_	-	21.21 (d)	19.79 (t)	43.7
**13**	AX _2_	29.70 (t)	-	27.95 (d)	60.7
**14**	AX _2_	26.73 (t)	-	15.46 (d)	51.0
**15**	AX _2_	30.19 (t)	-	27.71 (d)	60.7
**16**	AX _2_	25.93 (t)	-	15.55 (d)	51.0

The shifts, multiplicities, and coupling constants of the phosphazenes in the ^13^ C and ^1^ H spectra were evaluated (Tables S1 and S2). The expected carbon peaks were interpreted using the ^13^ C spectra. The carbon peaks of the phenyl rings, C1–C4, were assigned between 125.27 and 138.97 ppm (Table S1). The peaks of the OCH _2_ carbons of the spiro rings were between 68.23 and 63.00 ppm. In addition, the signals of the benzylic carbons (PhCH2) of the products, including the 6-membered spiro rings (4, 6, 8, 10, 12, 14, and 16), were shifted further downfield than those of the 5-membered rings (3, 5, 7, 9, 11, 13, and 15), as observed previously [28,29]. The same phosphorus atoms, to which 2 heterocyclic groups had bonded, showed 2 groups of NCH _2_ , NCH _2_ CH _2_ , NCH _2_ CH _2_ CH _2_ , and OCO peaks, with small separations in the ^13^ C spectra. Additionally, coupling constants between the C1 and P atoms ( ^3^ J _*PC*_ ) emerged in all of the phosphazenes, and the average ^3^ J _*PC*_ value was 8.6 Hz. Coupling constants ( ^2^ J _*PC*_ ) of the N C H _2_ spiro-groups of the products, including the 5-membered spiro rings (3, 5, 7, 9, 11, 13, and 15), were considerably large. The average ^2^ J _*PC*_ value was 12.3 Hz.

The expected proton signals of the phosphazenes were determined from the ^1^ H spectra (Table S2). The chemical shifts of the aromatic protons (H2, H3, and H4) were in the range of 7.43–7.21 ppm. The δ -values of the PhCH _2_ N protons of the monospirocyclotriphosphazenes (3–12) appeared to be in the range of 4.15–3.86 ppm, like the doublets, due to vicinal coupling with the ^31^ P nucleus. However, the corresponding protons of the dispirocyclotriphosphazenes (13–16) had an ABX spin system due to vicinal and geminal couplings with the P atoms and geminal protons, indicating that the 2 geminal protons of the PhCH _2_ N were diastereotopic. In addition, the ^3^ J _*PH*_ data of the phosphazenes possessing 5-membered spiro rings (3, 5, 7, 9, 11, 13, and 15) were slightly smaller than those of the 6-membered rings (4, 6, 8, 10, 12, 14, and 16). The average ^3^ J _*PH*_ couplings were 8.1 and 8.3 Hz for the 5- and 6-membered spirophosphazenes. Furthermore, δ -shifts of the NCH _2_ and OCH _2_ protons of the spiro-precursors were assigned in the ranges of 3.26–2.89 ppm and 4.43–4.21 ppm.

Asymmetric ν PN vibrations of the trimeric cyclophosphazenes were assigned in the range of 1200–1172 cm ^-1^ in the IR spectra [30]. The aromatic C-H bands were between 3040 cm ^-1^ and 3100 cm ^-1^ . Moreover, the asymmetric ν PCl _2_ vibrations of the partly substituted tetra (3 and 4) and dichloro phosphazenes (13–16) were determined in the range of 562–576 cm ^-1^ . These peaks were not observed in the IR spectra of the tetrakis-substituted monospirophosphazene derivatives (5–12).

### 2.3. X-ray structures of compounds 5 and 14

**Figure 1:**
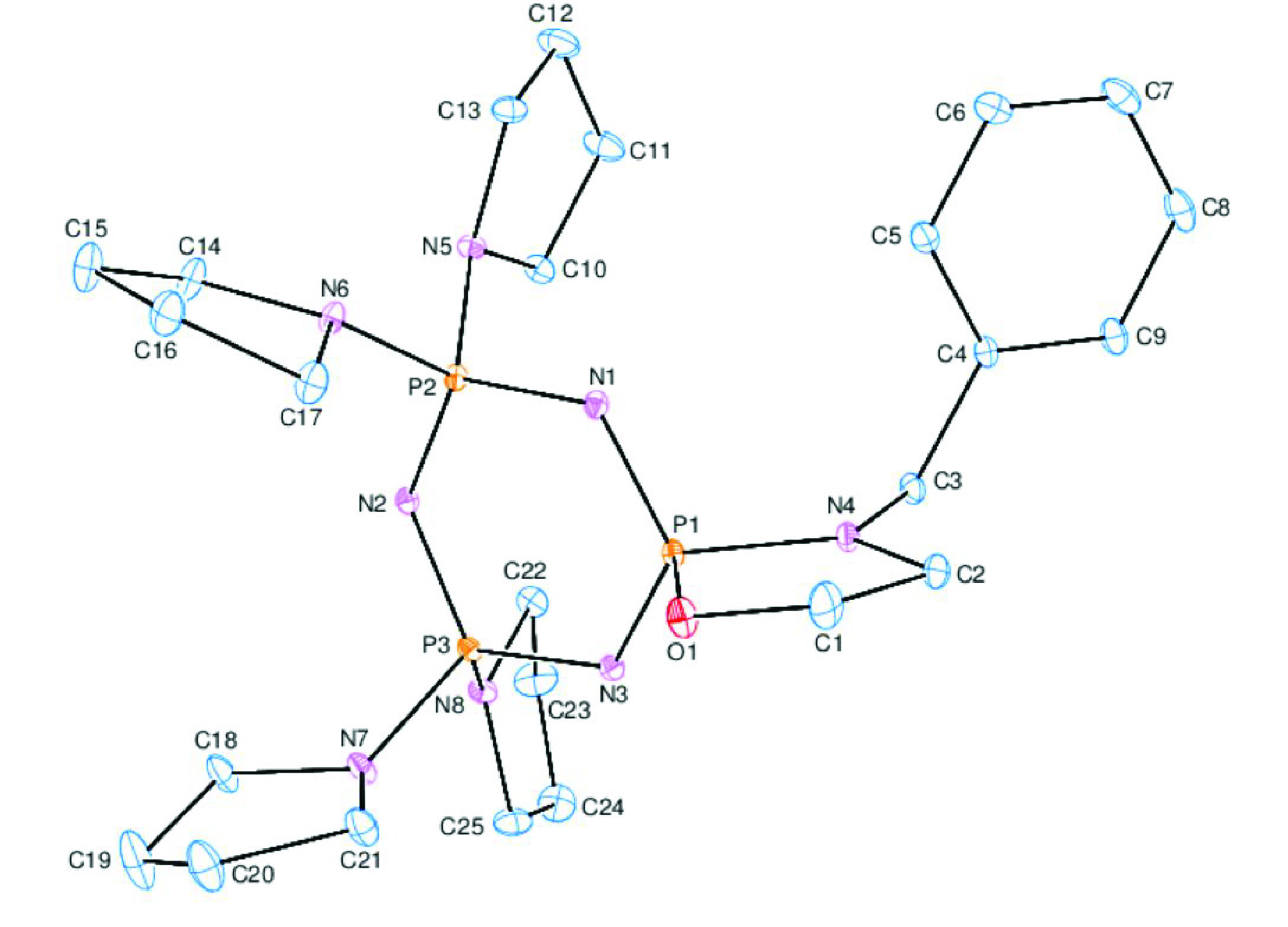
ORTEP-3 drawing of compound5with the atom-numbering scheme. Displacement ellipsoids are drawn at the 30% probability level.

**Figure 2:**
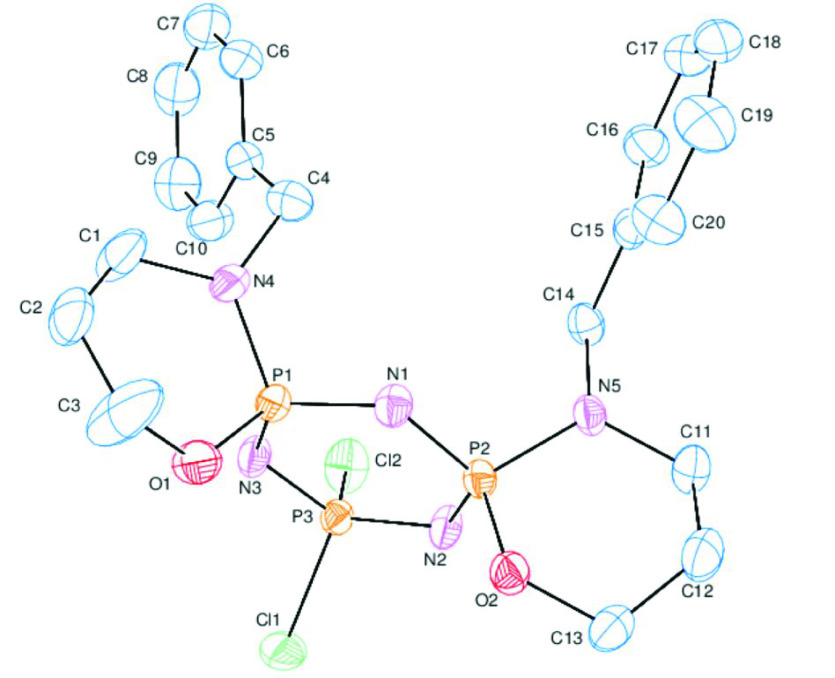
ORTEP-3 drawing of compound14with the atom-numbering scheme. Displacement ellipsoids are drawn at the 30% probability level.

The crystal structures of compounds 5 and 14 were assigned crystallographically. The crystallographic data of the new products are tabulated in Table 2, and ORTEP diagrams with suitable atom numbering are illustrated in Figures 1 and 2, respectively. Compound 14 had a cis-conformation with respect to the crystallographic results. The trimeric phosphazene skeletons, P1/N1/P2/N2/P3/N3, of compounds 5 and 14 [Figure S1; ϕ _2_ = 71.41(2)◦ , θ _2_ = 153.2(8)◦ (for 5); Figure S2; ϕ _2_ = 5.80(1)◦ , θ _2_ = 86.9(1)◦ (for 14)] were in twisted forms, indicating total puckering amplitudes Q _T_ of 0.120(2) Å (for 5) and 0.145(2) Å (for 14) [31]. As expected, the 5-membered spiro-ring of 5 was in envelope conformation (Figure S3). Both of the spiro-rings of 14 were in chair conformations (Figure S4). Compound 14 crystallized in the P bca space group. The absolute configurations of the P1 and P2 atoms of 14 were determined as S and R, respectively. The shapes of the phosphazene skeletons in 5 and 14 with torsion angles are given in Figure S5, displaying the pseudo mirror plane running from the P1-N1 atoms of the cyclotriphosphazene ring.

**Table 2:** Crystallographic data for compounds5and14

	5	14
Empirical formula	C _25_ H _43_ N _8_ P _3_ O	C _20_ H _26_ N _5_ P _3_ O _2_ Cl _2_
Fw	564.60	532.27
Crystal system	Triclinic	Orthorhombic
Space group	P -1	P bca
a(Å)	10.0618(3)	10.1221(3)
b(Å)	10.1133(3)	18.2196(4)
c(Å)	14.3780(4)	26.5541(5)
α(◦)	91.355(2)	90
β(◦)	96.254(3)	90
γ(◦)	92.965(2)	90
V(Ao3)	1451.79(7)	4897.1(2)
Z	2	8
μ(cm ^-1^ )	0.239 (Mo Kα)	0.489 (Mo Kα)
ρ(Calc.) (g cm ^-1^ )	1.292	1.444
Number of reflections Total	21354	55074
Number of reflections Unique	5111	4334
Rint	0.0281	0.0615
2θmax(◦)	50.06	50.14
Tmin/Tmax	0.675/0.765	0.8671/0.9391
Number of parameters	334	289
R [F2>2σ(F2)]	0.0522	0.0447
wR	0.1421	0.1051

Table 3 lists the characteristic bond angles and lengths of 5 and 14. The exocyclic PN bond lengths were 1.642(2) Å (for 5), and 1.633(3) and 1.637(2) Å (for 14), while the endocyclic PN bond lengths were in the ranges of 1.574(2)–1.603(2) Å (for 5) and 1.554(3)–1.612(3) Å (for 14). The exocyclic PN bonds were longer than the endocyclic PN bonds. Furthermore, the endocyclic PN bonds were shorter than the PN single bond reported in the literature [32], indicating that the phosphazene ring had nearly double the bond character. Furthermore, regular variations of the bonds in 5 and 14 were also observed, with the distances from P3: P3–N3 ≈ P2–N1 > P3–N2 ≈ P2–N2 > P1–N1 ≈ P1–N3 and P3–N3 ≈ P3–N2 ? P2–N1 ≈ P1–N1 ? P2–N2 ≈P1–N3.

**Table 3:** Selected bond lengths (Å) and angles (deg) for compounds5and14.

	5	14
P1–N1	1.574(2)	1.579(3)
P1–N3	1.584(2)	1.606(3)
P2–N1	1.600(2)	1.574(3)
P2–N2	1.594(2)	1.612(3)
P3–N2	1.588(2)	1.556(3)
P3–N3	1.603(2)	1.554(3)
P1–N4	1.641(2)	1.633(3)
P1–O1	1.607(2)	1.565(2)
P2–N5	-	1.637(2)
P2–O2	-	1.572(2)
N1–P1–N3 (α)	116.27(12)	114.20(13)
N1–P2–N2 (γ)(αfor14)	115.81(12)	114.95(13)
N2–P3–N3 (γ)	115.85(11)	121.20(14)
P1–N1–P2 (β)	123.98(14)	125.54(16)
P2–N2–P3 (δ)	123.03(13)	120.57(16)
P1–N3–P3 (β)	124.43(10)	121.48(16)
N4–P1–O1 (α’)	93.93(11)	103.03(14)
N5–P2–O2 (α’)	-	103.43(12)

The endocyclic NPN (α) angles of tetrakis-pyrrolidino monospiro (5) and cis-dispirophosphazenes (14) [116.27(12)◦ (for 5), and 114.20(13)◦ and 114.95(13)◦ (for 14)] were narrow with respect to the values of N _3_ P _3_ Cl _6_ , a standard compound [33]. The β and δ (PNP) angles of 5 and 14 were in the ranges of 123.03(13)◦ – 124.43(10)◦ (for 5) and 120.57(16)◦ –125.54(16) (for 14) (Table 3). Additionally, it was concluded that the exocyclic NPO bond angles of these phosphazenes [93.93(11)◦ (for 5), 103.03(14)◦ and 103.43(12) (for 14)] were considerably narrow when compared with N _3_ P _3_ Cl _6_ . All of the variations in the bond angles and lengths may be attributed to the steric interactions between the bulky groups and the negative hyperconjugation [34]. When compared with the literature findings, it was clear that the variations in the bond angles and length variations of 5 and 14 were in good agreement with the previously reported data [35].

In addition, the packing diagrams of 5 and 14 are presented in Figures S6 and S7, where the compounds are stacked through the a-axis direction.

### 2.4. Antimicrobial activity of the compounds

The antibacterial activities of 3–12 and 15 were evaluated against pathogenic bacteria, including 5 strains of G(+) bacteria (E. hirae, E. faecalis, B. subtilis, B. cereus, and S. aureus) and 6 strains of G(–) bacteria (2 E. coli and K. pneumoniae, S. typhimurium, P. vulgaris, and P. aeruginosa) using the agar well diffusion method. Evaluation of the antibacterial activities of these compounds is listed in Table S3. The results revealed that compounds 3, 6, 7, 8, 9, 11, 12, and 15 were effective in suppressing the microbial growth of pathogenic bacteria with variable potency [compound 3 was active against S. typhimurium G(–) and P. vulgaris G(–); compound 6 against S. aureus G(+) and P. aeruginosa G(–); compound 7 against P. aeruginosa G(–); compound 9 against S. aureus G(+) and P. aeruginosa G(–); compound 10 against P. vulgaris G(–) and S. aureus G(+); compound 11 against E. hirae G(+), P. aeruginosa G(–), and P. vulgaris G(–); and compound 15 against E. coli ATCC 25922 G(–), K. pneumoniae, P. vulgaris G(–), P. aeruginosa G(–), S. aureus G(+), and B. subtilis G(+)]. Compounds 10 and 11 were the most effective at inhibiting microbial growth of the tested bacteria at a concentration of 2500 μM, whereas compound 15 was effective against several bacterial species. Compounds 6–8 were effective against P. aeruginosa. The compounds showed variable antimicrobial activities against fungal strains. Findings of the antimicrobial activities of the products indicated that E. faecalis was the bacterium most resistant to the compounds, followed by E. coli, whereas S. aureus, S. typhimurium, and P. aeruginosa were the bacteria most susceptible to the compounds. Furthermore, compounds 10, 11, and 15 were the most active and exhibited strong antibacterial effects against pathogenic bacteria. It was understood that the pyrrolidino (6), piperidino (9 and 10), and DASD-substituted benzyl-pendant-armed monospirophosphazenes had activity against G(+) bacteria, while compounds possessing chloro (3 and 4) and morpholino (7 and 8) had no activity against G(+) bacteria. It appeared that the tetrachloro monospiro (3) and dichloro dispiro (15) compounds with 5-membered spiro-ring(s) had activity against G(–) bacteria, S. typhimurium, P. vulgaris, E. coli ATCC 25922, and K. pneumoniae. According to the minimum inhibitory concentration (MIC) and minimal bacterial concentration (MBC) values, tetrakis-piperidino phsophazene (9) was more effective than ampicillin and chloramphenicol against B. cereus (G+). Additionally, the tetrakis-pyrrolidino (6), morpholino (7 and 8), and DASD-substituted (11) phosphazenes were very effective against G(–) bacteria (P. aeruginosa and P. vulgaris).

In addition, compounds 4, 7, 8, 9, 10, and 12 showed strong antifungal activity. They exhibited inhibitory effects against C. krusei fungus, whereas compound 10 was also effective against C. tropicalis. The substituents, i.e. chloro (4, six-membered spiro ring), morpholino (7 and 8), piperidino (9 and 10), and DASD (12, six-membered spiro ring), with the exception of pyrrolidino (5 and 6) and two-benzyl-pendant-armed dispiro (15), played an important role in the activity of the benzyl-pendant-armed monospirophosphazenes.

Experiments were performed to determine their MICs and MBCs against strains of S. typhimurium, P. vulgaris, S. aureus, P. aeruginosa, E. hirae, B. cereus, C. tropicalis, and C. krusei (Tables 4 and 5). The MICs and MBCs were in the range of 125–500 μM.

**Table 4:** MIC values of compounds 3, 4, and 6–12 on different bacterial and fungal species (in μM) (Amp: ampicillin, C: chloramphenicol (antibacterial), and Keto: ketoconazole (antifungal) used as a control; “-”: the compounds did not cause any growth inhibition; NS: not studied).

Test organisms/compounds	3	4	5	6	7	8	9	10	11	12	Amp	C	Keto
*E. hirae*	-	-	-	-	-	-	500	-	<19.5	156	NS
*S. aureus*	-	-	250	-	-	-	-	-	<19.5	156	NS
*P. aeroginosa*	-	-	125	125	-	-	125	-	>2500	>2500	NS
*P. vulgaris*	250	-	-	250	-	-	250	-	1250	1250	NS
*S. typhimurium*	250	-	-	-	-	-	-	-	<19.5	156	NS
*B. cereus*	-	-	-	-	125	-	-	-	156	156	NS
*C. tropicalis*	-	-	-	-	-	250	-	-	NS	NS	78
*C. krusei*	-	250	125	125	125	125	-	250	NS	NS	< 19.5

**Table 5:** MBC and MFC values of compounds 3, 4, and 6–12 on different bacterial and fungal species (in μM).

Test organisms/compounds	3	4	5	6	7	8	9	10	11	12	Amp	C	Keto
*E. hirae*	-	-	-	-	-	-	-	1000	39	2500	NS
*S. aureus*	-	-	500	-	-	-	-	-	< 19.5	<19.5	NS
*P. aeroginosa*	-	-	250	125	250	-	-	125	>2500	> 2500	NS
*P. vulgaris*	250	-	-	-	250	-	-	250	>2500	2500	NS
*S. typhimurium*	500	-	-	-	-	-	-	-	<19.5	<19.5	NS
*B. cereus*	-	-	-	-	-	125	-	-	2500	2500	NS
*C. tropicalis*	-	-	-	-	-	-	500	-	NS	NS	1250
*C. krusei*	-	500	-	250	250	250	250	-	NS	NS	156

Consequently, the antibacterial and antifungal activity results observed in this study were comparable with the literature data. In the literature, pyrrolidino- and DASD-substituted cyclotriphosphazenes containing ferrocenyl/arylspirocyclic pendant arms also had activity against some G(+) and G(–) bacteria and fungi [8,25,26,28,36]. In the present study, the chloro-, pyrrolidino-, morpholino-, piperidino-, and DASD-substituted cyclotriphosphazenes had different antibacterial and fungal activities, as listed in Tables 4 and 5. The antibacterial and antifungal activities of the mono- and dispirocyclotriphosphazene derivatives with benzyl pendant arms could have been due to the formation of hydrogen bonding, dipole-dipole interactions between the DNA of the bacteria and fungi, or the phosphazene derivatives used in this study. On the other hand, the substituents (chloro, pyrrolidino, DASD, piperidino, and morpholino), the number of members, and the conformations of the spiro rings may also have played an important role in the activity.

### 2.5. Interactions of DNA with compounds 3–12 and 15

**Figure 3:**
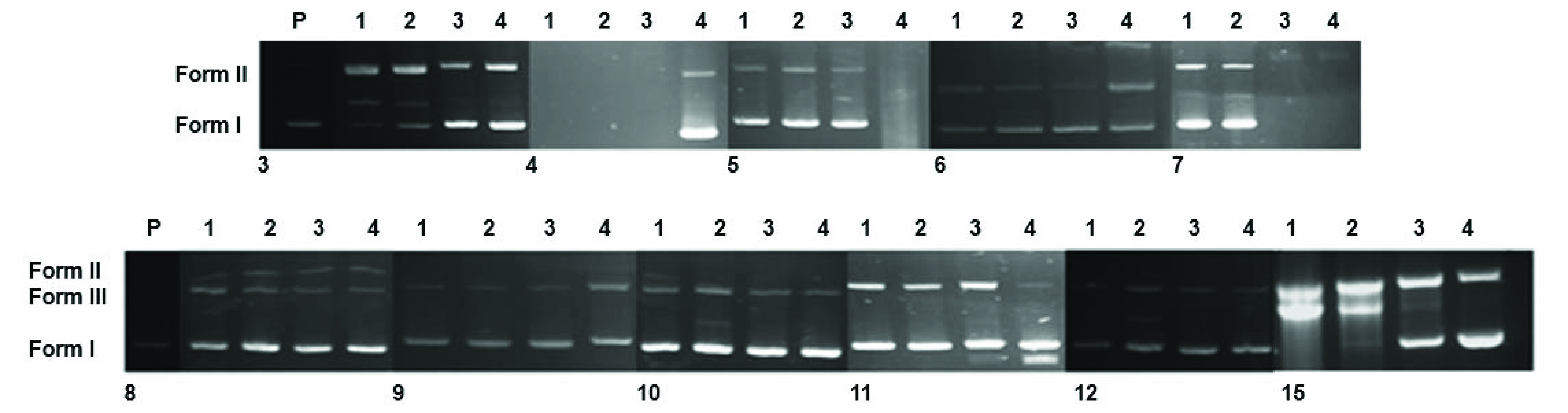
Electrophoretograms of the incubated mixture of pBR322 plasmid DNA and varying concentrations of compounds 3–12 and 15. Lane P: Untreated plasmid DNA. Plasmid DNA of Lanes 1 to 4 interacted with decreasing concentrations of the compounds. Concentrations for the compounds: Lane 1: 2500, Lane: 1250, Lane 3: 625, and Lane 4: 312.5 μM.

In the present study, 2 DNA bands, namely Forms I and II, appeared for both untreated and treated pBR322 with decreasing concentrations of compounds 6–15 (except for compounds 3, 4, and 15) (Figure 3). In compound 15, a faint, almost coalesced band was observed at 2500 μM (Lane 1), and when the concentrations of the products decreased, the mobility of the bands of Forms I and II increased. The change was less significant with compound 3 at three concentrations, but Form I disappeared at the highest concentration. With compound 4, Forms I and II disappeared at three high concentrations. This was the most damaging compound for the DNA when compared to compound 15. With 15, the separations between the bands were the smallest at 2500–1250 μM (Lanes 1 and 2), below which the separations were larger (Lanes 3 and 4). The presence of a coalesced band suggested a change in the conformation of the DNA of Form I, from the supercoiled Form I to the negative form and from the negative 1 to the positive form [37]. In all of the other compounds, two bands corresponding to Forms I and II were observed in all of the lanes, and a single faint band could be seen in Lanes 2–4 (for 8) and Lane 4 (for 11), corresponding to concentrations of 1250, 625, and 312.5 μM (for 8) and 312.5 μM (for 11), respectively. No bands were observed in Lanes 7 and 8. These bands could have been due to the induced changes in the DNA conformations.

## 3. Experimental

### 3.1. Reagents used for synthesis

The aliphatic amines (Fluka), ethanolamine, 3-amino-1-propanol, benzaldehyde, and trimer were purchased from Sigma-Aldrich (St. Louis, MO, USA). They were used without further purification. All reactions were followed by TLC using Merck DC Alufolien Kieselgel 60 B254 sheets (Darmstadt, Germany). Merck Kieselgel 60 (230–400 mesh ATSM) silica gel was used for the column chromatography.

### 3.2. Physical measurements

Microanalytical data were obtained using a LECO CHNS-932 analyzer (St. Joseph, MI, USA). The NMR spectra were supplied on a Bruker DPX FT-NMR (500 MHz) spectrometer (Billerica, MA, USA) (SiMe4 as internal and 85% H3 PO4 as external standards). The spectrometer was equipped with a 5-mm PABBO BB inverse-gradient probe. Standard Bruker pulse programs [38] were used. The IR spectra were obtained on a Mattson 1000 IR spectrometer in KBr disks and were given as cm ^-1^ . APIES mass analyses were made on a Waters 2695 Alliance Micromass ZQ spectrometer (Milford, MA, USA).

### 3.3. Preparations of the compounds

Compounds 1–6 were synthesized according to the literature [27], whereas compounds 1 and 2 were synthesized in ethanol and phosphazenes 3–6 were synthesized in THF.

Compound 3: Yield: 2.32 g (68%; lit. 44%). mp: 112 ◦ C (lit. 114 ◦ C).

Compound 4: Yield: 2.29 g (65%; lit. 55%). mp: 110 ◦ C (lit. 110 ◦ C).

Compound 5: Yield: 0.86 g (66%; lit. 34%). mp: 188 ◦ C (lit. 188 ◦ C).

Compound 6: Yield: 0.94 g (71%; lit. 45%). mp: 138 ◦ C (lit. 138 ◦ C).

#### 3.3.1. General procedure used for synthesis of the fully-substituted monospirocyclotriphosphazenes (7–12)

The solution of heterocyclic amines dissolved in THF (50 mL) was added to the solution of tetrachloromonospirocyclotriphosphazene derivatives in dry THF (100 mL) in the presence of triethylamine (Et3 N). The reactions were refluxed for 24 h by stirring. The crude compounds were separated using column chromatography [toluene and THF (3:1) as eluent]. The phosphazene derivatives were crystallized from petroleum ether (60–80 ◦ C).

Information on synthesis of the fully substituted monospirocyclotriphosphazenes (7–12) is given in Table 6.

**Table 6:** Information on the synthesis of the fully-substituted monospirocyclotriphosphazenes (7–12).

Tetrachloromonospiro cyclotriphosphazene				Heterocyclic amine						Et3 N (mL)	Product	Yield (%)	m.p. (◦C)	Rf toluene and THF (2:1)
3		4		Morp.		Pip.		DASD						
G	mmol	g	mmol	mL	mmol	mL	mmol	mL	mmol					
0.80	1.88	-	-	1.94	22.54	-	-	-	-	3	7	68 (0.79 g)	158	0.12
-	-	0.80	1.81	1.88	21.82	-	-	-	-	3	8	70 (0.81 g)	139	0.16
0.80	1.88	-	-	-	-	2.23	22.54	-	-	3	9	72 (0.84 g)	130	0.72
-	-	0.60	1.36	-	-	1.62	16.36	-	-	3	10	73 (0.63 g)	121	0.74
0.80	1.88	-	-	-	-	-	-	2.89	22.56	3	11	79 (1.24 g)	140	0.19
-	-	0.60	1.36	-	-	-	-	2.09	16.32	3	12	81 (0.95 g)	129	0.23

#### 3.3.2. General procedure used for synthesis of the cis- and trans-dispirocyclotriphosphazenes (13–16)

The mixtures of sodium (N-benzyl)aminopropanoxide and Et3 N in THF were added to a stirred solution of N _3_ P _3_ Cl _6_ in THF (50 mL), and stirred and refluxed for 30 h. After the precipitated triethylaminehydrochloride was filtered off, the solvent was evaporated. The three products were purified by column chromatography with toluene and THF (15:1). The first product was the tetrachloro monospirophosphazene derivative. The second product was the cis-dispirocyclophosphazene. The last product was the trans-dispirocyclic compound (15). The compounds were crystallized from petroleum ether (60–80 ◦ C) at room temperature.

Information on synthesis of the dispirocyclotriphosphazenes (13–16) is given in Table 7.

**Table 7:** Information on the synthesis of the dispirocyclotriphosphazenes (13–16).

Trimer		Comp. 1		Comp. 2		Et3N (mL)	Product	Yield (%)	m.p. (◦C)	Rf toluene Êand THF (2:1)
G	mmol	g	mmol	mL	mmol					
4.52	13.00	3.93	26.00	-	-	7.50	3	26 (1.46 g)		
							13	30 (1.97 g)	108	0.70
							15	37 (2.42 g)	114	0.49
3.16	9.09	-	-	3.00	18.18	9.00	4	25 (1.00 g)		
							14	32 (1.55 g)	118	0.72
							16	37 (1.79 g)	124	0.54

The IR, APIES-MS, and microanalytical data of the products (7–16) are tabulated in Table 8.

**Table 8:** IR, APIES-MS, and microanalytical data of compounds 7–16.

Compound	Elemental analyses (%) (Calc./Found)			APIES-MS (Ir %)		IR (ν, KBr, cm ^-1^ )
	C	H	N	Calc.	Found	
7 (P3N8C25H43O5)	47.77/47.74	6.90/7.07	17.83/17.45	629	630	3100 (C-H arom.), 2974 (C-H aliph; asymm.), 2902 (C-H aliph; symm.), 1196 (P=N asymm.)
8 (P3N8C26H45O5)	48.60/48.29	6.74/6.86	17.44/17.13	643	644	3100 (C-H arom.), 2976 (asymm.), 2904 (symm.) (C-H aliph), 1188 (asymm.)
9 (P3N8C29H51O)	56.12/56.42	8.28/8.03	18.05/17.76	621	622	3040 (C-H arom.), 2928 (asymm.), 2830 (symm.) (C-H aliph.), 1186 (asymm.) (P=N)
10 (P3N8C30H53O)	56.77/56.51	8.42/8.09	17.65/17.36	635	636	3080 (C-H arom.), 2972 (asymm.), 2924 (symm.) (C-H aliph.), 1190 (asymm.), (P=N)
11 (P3N8C37H59O9)	52.11/52.05	6.97/7.02	13.14/12.90	853	854	3100 (C-H arom.), 2972 (asymm.), 2902 (symm.) (C-H aliph), 1200 (asymm.) (P=N)
12 (P3N8C38H61O9)	52.65/52.41	7.09/7.16	12.93/12.83	867	868	3100 (C-H arom.), 2974 (asymm.), 2906 (symm.) (C-H aliph), 1200 (asymm.)
13 (C18H22N5P3Cl2O2)	42.90/42.81	4.40/4.52	13.90/13.74	503	504	3076 (C-H arom.), 2962 (asymm.), 2902 (symm.) (C-H aliph), 1182 (asymm.) (P=N), 572 (asymm.) (PCl)
14 (C20H26N5P3Cl2O2)	45.13/45.53	4.92/4.59	13.16/13.43	531	532	3088 (C-H arom.), 2968 (asymm.), 2852 (symm.) (C-H aliph), 1172 (asymm.) (P=N), 576 (asymm.) (PCl)
15 (C18H22N5P3Cl2O2)	42.90/42.77	4.40/4.67	13.90/13.80	503	504	3062 (C-H arom.), 2968 (asymm.), 2892 (symm.) (C-H aliph), 1176 (asymm.) (P=N), 566 (asymm.) (PCl)
16 (C20H26N5P3Cl2O2)	45.13/45.22	4.92/4.86	13.16/13.01	531	532	3098 (C-H arom.), 2928 (asymm.), 2906 (symm.) (C-H aliph), 1176 (asymm.) (P=N), 562 (asymm.) (PCl)

### 3.4. X-ray crystallography

Single crystals of compounds 5 and 14 were grown in acetonitrile at 296 K, and their crystallographic data were collected on a Bruker APEXII CCD area-detector diffractometer by Mo Kα . The multiscan absorption correction [39] applied data were processed by SHELX program packages [40,41] for solving and refining the structures, and the ORTEP-3 program [42] was used for the drawings. H atom positions were calculated geometrically at distances of 0.93 and 0.97 Å for methine and methylene, respectively, and refined using a riding model by applying the constraint of 1.2 Ueq (carrier atom) for the Uiso (H) values.

Determinations of the antimicrobial activities and MIC/MBC/MFC values of the phosphazenes as well as DNA interactions with the compounds were performed according to the method in the literature [43].

## 4. Conclusion

The present study focused on the syntheses of monospiro (3–12) and dispirocyclotriphosphazenes (13–16) with benzyl pendant arms. One of the most important aspects of this study was to separate the cis (13 and 14) and trans (15 and 16) isomers in pure form. The structures of 5 and 14 were determined using X-ray data. The spectral data ( ^1^ H, ^13^ C, and ^31^ P) showed that the structures of all of the compounds were in good agreement with the proposed formulae. The structures of 5 and 14 were symmetric in the solid state with respect to X-ray crystallography. The data showed that the absolute configuration of 14 was SR. In addition, the monospiro (3 and 4), cis (meso) (13 and 14), and trans (racemic) (15 and 16) cyclotriphosphazenes possessed prochiral, diastereotopic, and homotopic atoms. Additionally, most of the compounds were found to inhibit most of the antibacterial activities of bacteria, except for P. aeruginosa, S. aureus, and B. cereus. The antimicrobial activities and MIC values revealed that the most effective compounds were 6–8 and 11, and the bacterium most affected by the compounds was P. aeruginosa. The MBC and MFC values of compounds 3, 4, and 6–12 ranged from 250 to 1000 μM. DNA interaction studies of the cyclotriphosphazenes revealed that compounds 3, 4, and 15 had a strong effect on supercoiled DNA by cleavage of the double helix. In conclusion, the partly tetrachloro monospiro (3 and 4) and dichloro dispirocyclotriphosphazenes (13–16) could be useful starting compounds for the synthesis of the new organocyclotriphosphazenes as a chiral catalyst. Additionally, the tetrakis-substituted phosphazenes (5–12) are thought to be strong phosphazene bases that can be used as ligating ligands for some metal cations.

## Supplementary Materials

Listings of the ^13^ C (decoupled) and ^1^ H data of the phosphazenes (Tables S1 and S2), ring conformations, and packing diagrams of compounds 5 and 14 (Figures S1–S4, S6, and S7), shapes of the phosphazene rings in compounds 5 and 14 with torsion angles (Figure S5), and antimicrobial activities, determination of the MIC and MBC/MFC values, and DNA-phosphazene interactions (Section S1) are provided as supplementary information. Crystallographic data for compounds 5 and 14 reported in this paper were deposited with the Cambridge Crystallographic Data Centre, CCDC Nos. 1941517 (for 5) and 1941518 (for 14). Copies of the data may be supplied through application to CCDC, 12 Union Road, Cambridge CB2 1EZ, UK. (fax: +44 1223 336033 or e-mail: deposit@ccdc.cam.ac.uk or at http://www.ccdc.cam.ac.uk).
